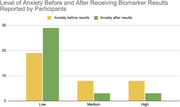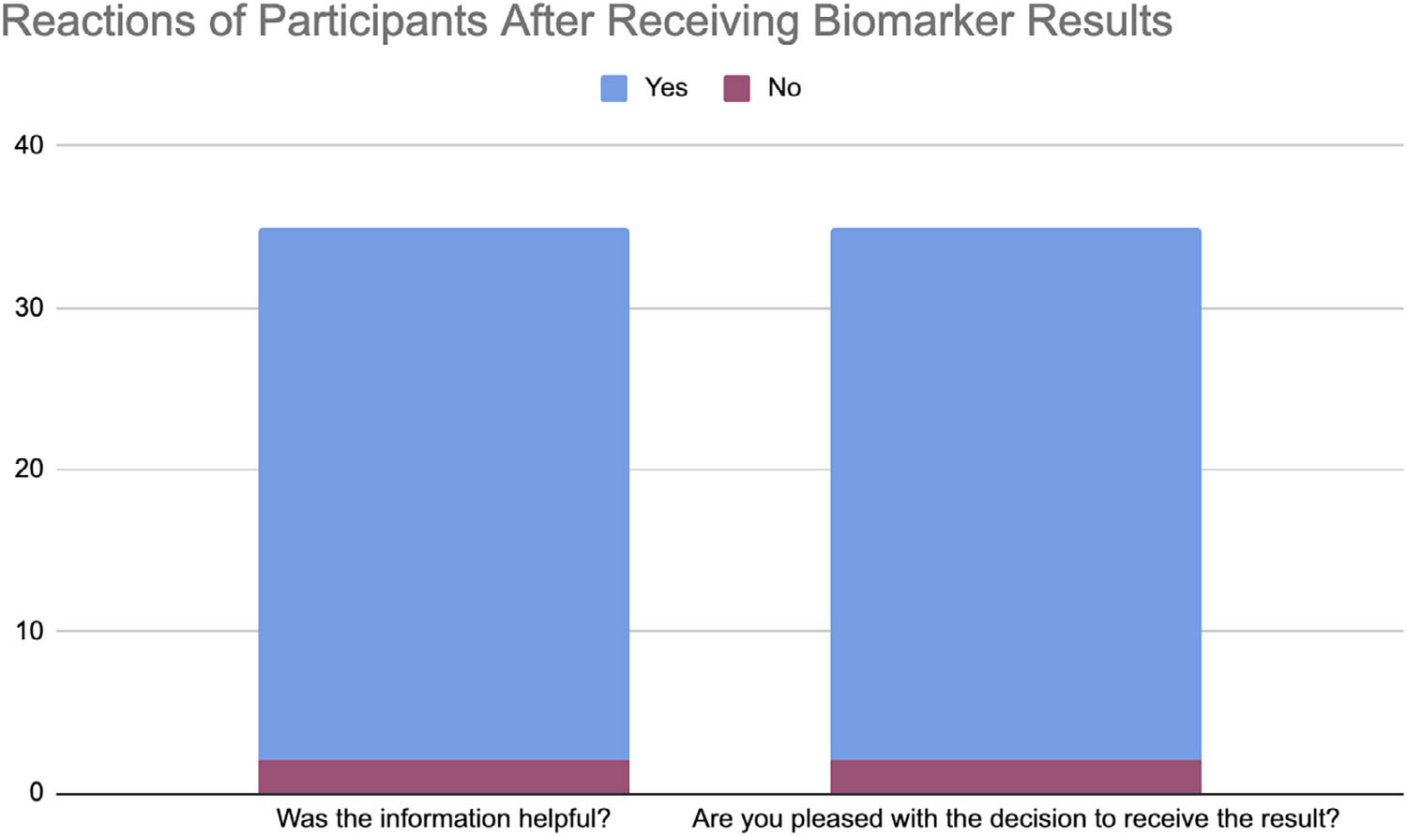# Process and metrics surrounding disclosure of genetic and biomarker results in the Duke University/University of North Carolina Alzheimer’s Disease Research Center (Duke/UNC ADRC) cohort

**DOI:** 10.1002/alz.089975

**Published:** 2025-01-09

**Authors:** Inbal Mayan, Heather Whitson, Heidi L Roth, Kim G Johnson

**Affiliations:** ^1^ Duke University, Durham, NC USA; ^2^ UNC, Chapel Hill, NC USA; ^3^ Neurology Department, Duke University Medical Center, Durham, NC USA

## Abstract

**Background:**

Increasingly, research participants in Alzheimer’s studies are seeking disclosure of research results. However, there are no guidelines for result disclosure and specifically, few studies report on their biomarker disclosure procedure. The Duke/UNC ADRC seeks to disclose research results to participants in a standardized fashion with high participant satisfaction.

**Method:**

The Duke/UNC ADRC cohort consists of participants aged 25‐80. Uniform Data Set (UDS) neuropsychological testing, MRI, APOE testing, CSF biomarkers including amyloid beta 42/40 (abeta 42/40), phospho tau (p‐tau) and total tau (t‐tau) are measured in the initial visits. A formal consensus panel confers a clinical and pathological diagnosis based on review of UDS and biomarker results. Participants receive their results during a telehealth appointment. With a standard slide presentation, the clinician educates participants about risk factors, biomarker significance and methods of clinical diagnosis. The clinician presents each participant’s unique results of APOE testing, CSF testing including amyloid and tau and consensus panel diagnosis. The conversation lasts about 25 minutes on average and participants are sent a survey through REDCap that consists of 5 questions. The post‐results survey, which takes 1‐2 minutes to complete, assesses anxiety levels, participant satisfaction and gives participants an option to seek more information.

**Result:**

Results were given to 50 participants. The post results survey completion rate was over 70% with 35 participants completing the survey. 94% (33/35) of participants were pleased with the decision to receive the results and reported the information helpful. Anxiety levels declined after receiving the results. While 46% (16/35) of participants reported medium/high level of anxiety before receiving results, only 17% (6/35) reported medium/high level of anxiety after receiving results.

**Conclusion:**

We show high participant satisfaction and low anxiety levels when receiving genetic and biomarker results. The process we developed shows promise to disclose biomarker information to participants in a standardized fashion, while providing education and sought after information about genetic and biomarker status.